# Supplementary material for: Transient growth factor expression via mRNA in lipid nanoparticles promotes hepatocyte cell therapy in mice
Source: Nat Commun. 2024 Jun 12;15:5010. doi: 10.1038/s41467-024-49332-8 (PMC11169405; doi:10.1038/s41467-024-49332-8)
Supplement: Supplementary file 1 — Supplementary Information [file 41467_2024_49332_MOESM1_ESM.pdf]

Smith et al.,

Supplementary Information:

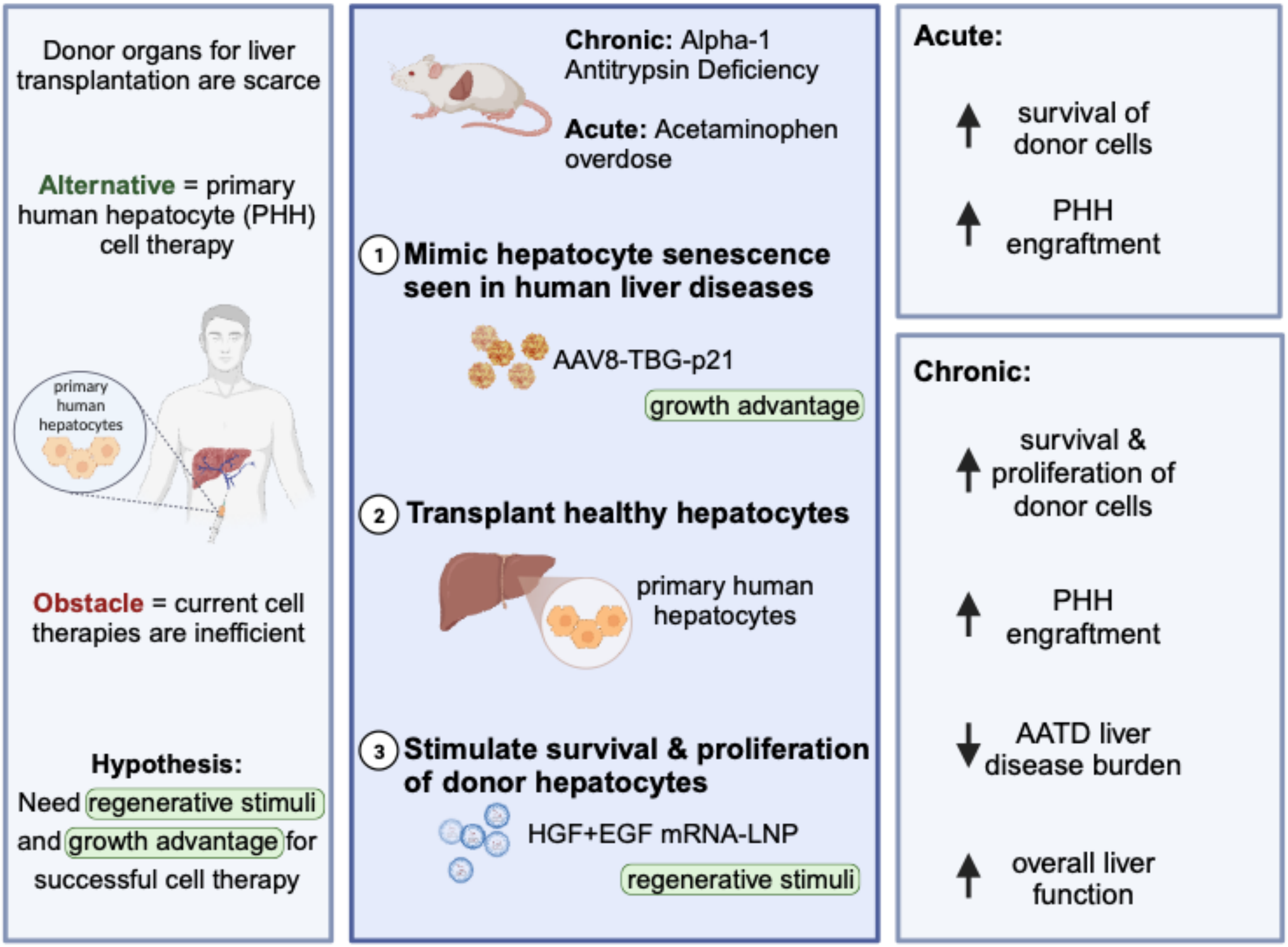

Supplementary Fig. 1 | Graphical Abstract. Liver transplantation remains the standard of care for patients with end stage liver disease, however, donor organs are always in limited supply. Alternatively, transplanting primary human hepatocytes (PHH) holds promise for restoring liver function, but with current hepatocyte cell therapies engraftment efficiency is low and long-term benefits are limited. The notion in the field is that to harness effective liver cell therapies, there must be regenerative stimuli and a growth advantage for donor cells. In two mouse liver disease models, one chronic and one acute, we mimic human liver diseases by compromising host hepatocyte proliferation by expressing p21. In these models, we demonstrate that transient, robust expression of human hepatocyte growth factor (HGF) and epidermal growth factor (EGF) in the liver delivered via nucleoside-modified mRNA in lipid nanoparticles (mRNA-LNP) significantly enhances PHH survival, proliferation, and engraftment. Importantly, in the chronic injury model PHH engraftment is sufficient to reduce disease burden and improve overall liver function by 5 weeks. This innovative approach may overcome the current barriers to translating hepatocyte cell therapies, primary or stem cell derived, to the clinic. Supplementary Fig. 1 Created with BioRender.com released under a Creative Commons Attribution-NonCommercial-NoDerivs 4.0 International license Created with BioRender.com.

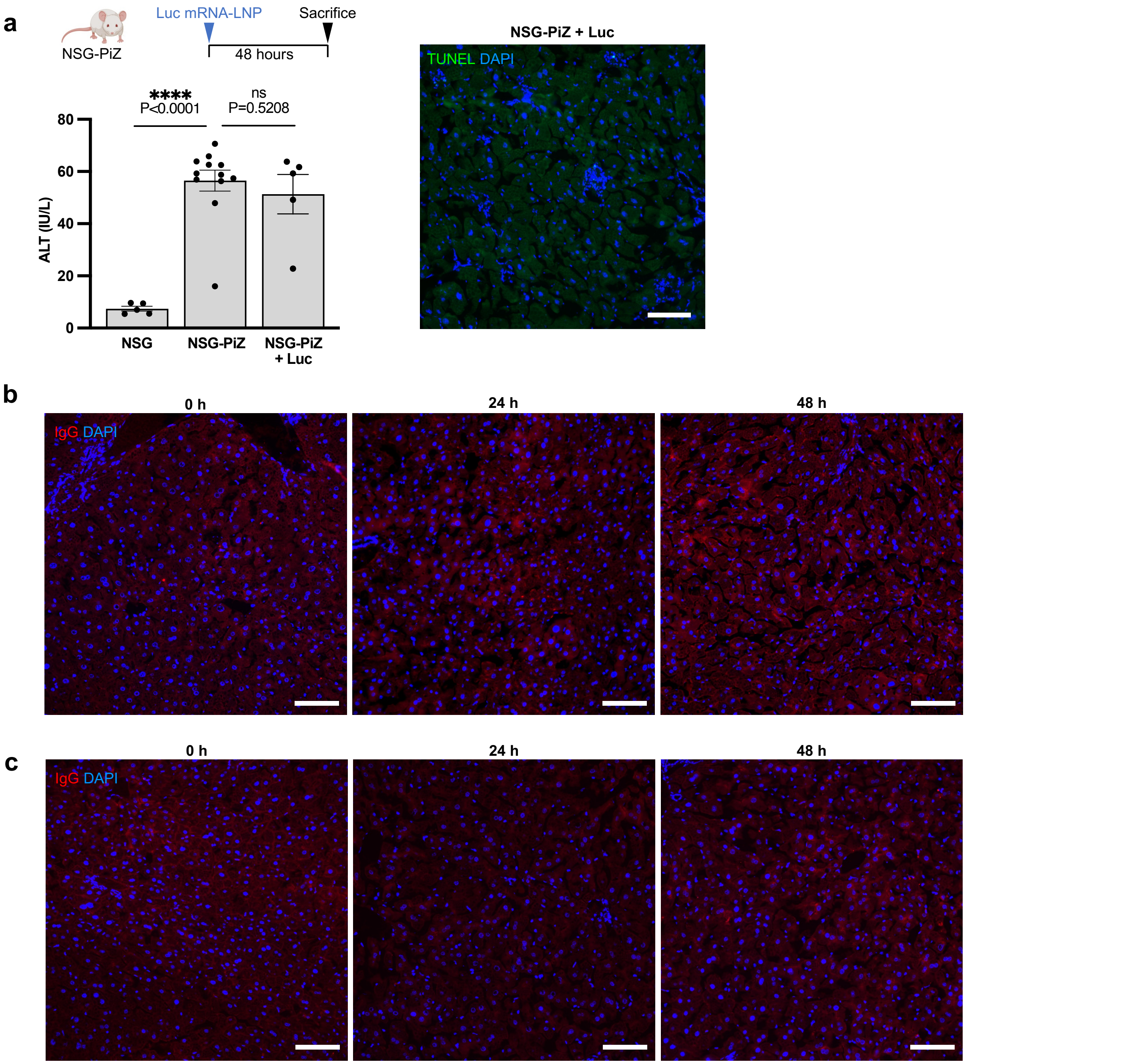

Supplementary Fig. 2 | mRNA-LNP do not induce hepatotoxicity & isotype control staining for HGF and EGF antibodies. a NSG-PiZ mice were injected with control Luc mRNA-LNP and sacrificed 48 hours later. Liver enzyme alanine aminotransferase (ALT) levels in serum were measured via kinetic spectrophotometric assay. ALT levels shown in comparison to age-matched untreated NSG and NSG-PiZ controls. Representative images of immunofluorescence staining for TUNEL on liver tissue of Luc mRNA-LNP injected mice. For NSG n=5 mice, for NSG-PiZ n=12 mice, for NSG-PiZ + Luc n=5 mice. Each dot represents one mouse, error bars = SEM, P values were calculated by unpaired two-sided student's t test, ns P > 0.05, * P ≤ 0.05, ** P ≤ 0.01, *** P ≤ 0.001, **** P ≤ 0.0001, scale = 100 μm. **b** Representative images of immunofluorescence staining for the HGF antibody isotype control on liver tissue of untreated NSG-PiZ mice and 24 hours or 48 hours after IV administration of HGF+EGF mRNA-LNP. **c** Representative images of immunofluorescence staining for the EGF antibody isotype control on liver tissue of untreated NSG-PiZ mice and 24 hours or 48 hours after IV administration of HGF+EGF mRNA-LNP. For panels **b,c**: n=3 mice per group, scale = 100 μm. Source data are provided as a Source Data file. Supplementary Fig. 2, Panel a Created with BioRender.com released under a Creative Commons Attribution-NonCommercial-NoDerivs 4.0 International license Created with BioRender.com.

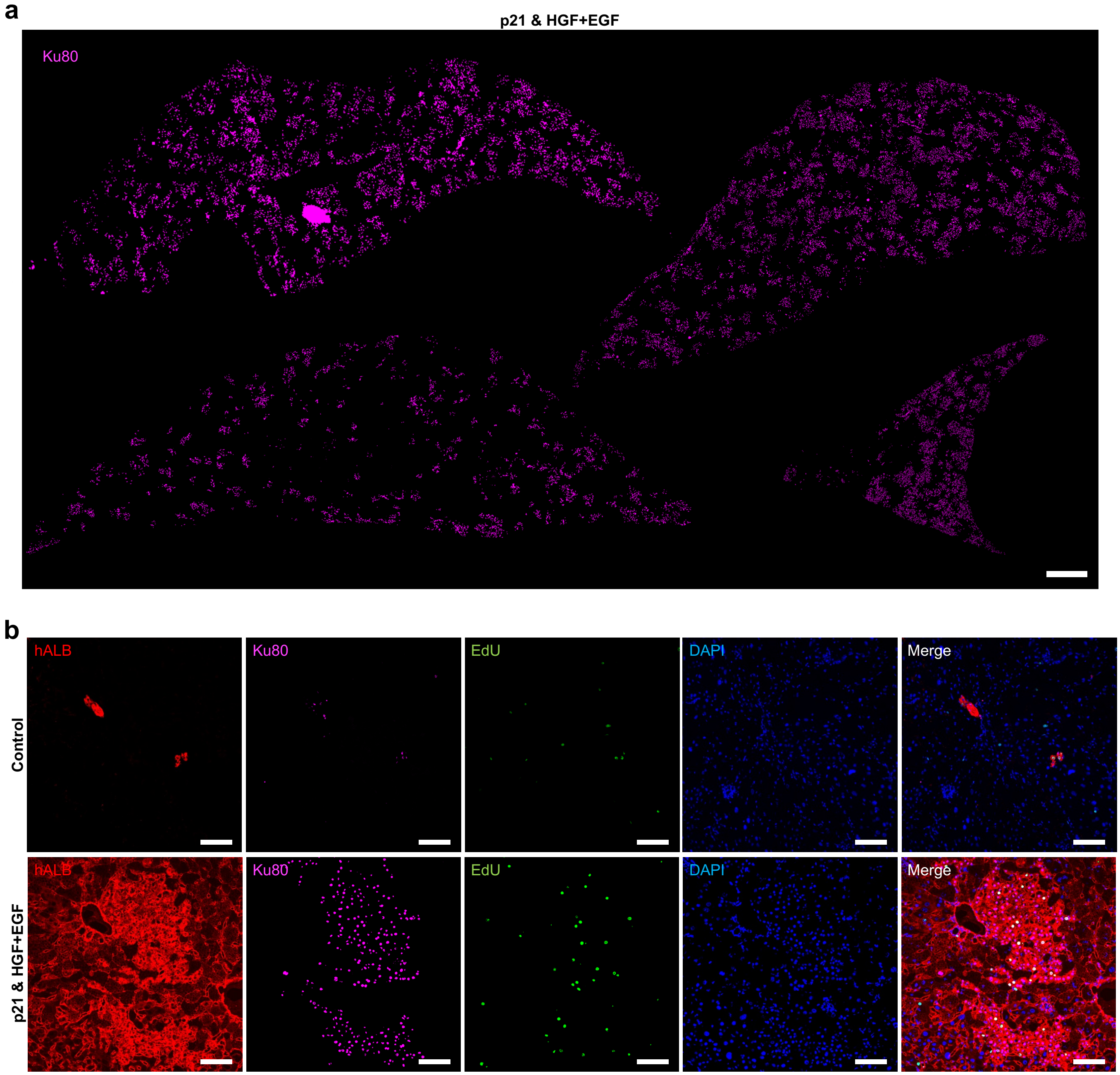

Supplementary Fig. 3 | HGF+EGF mRNA-LNP treatments lead to sustained and robust engraftment of PHHs in p21/NSG-PiZ mice. a Representative images of Ku80 immunofluorescence stain on liver sections from the experimental group 5 weeks post transplantation. One representative lobe is shown from each sample, various liver lobes are highlighted. n=5 mice, scale = 1000 μm. **b** Representative images of hALB/Ku80/EdU immunofluorescence stain on liver sections from experimental group 5 weeks post transplantation. Split and merged channels are shown. n=5 mice, scale = 100 μm.

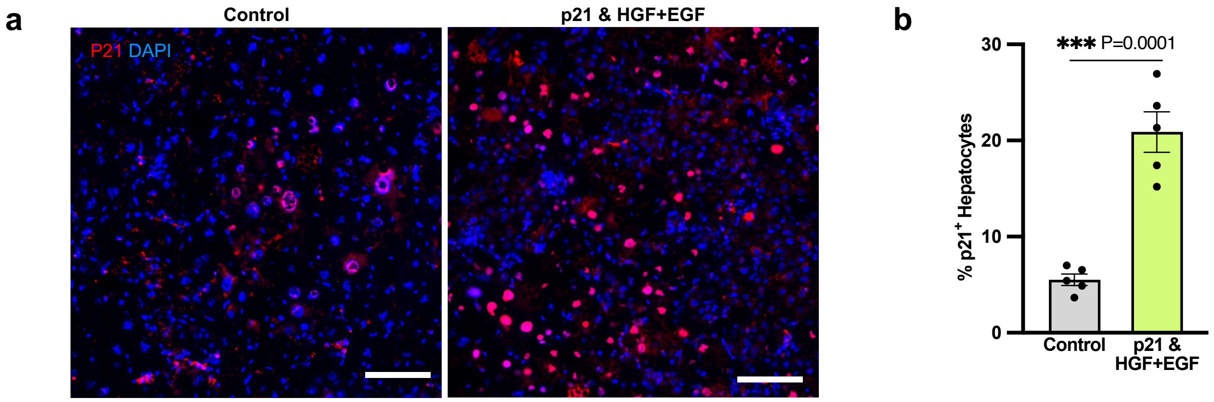

Supplementary Fig. 4 | p21 expression in mice 5 weeks post transplantation. a Representative images of immunofluorescence staining for p21 in transplanted NSG-PiZ livers 6 weeks after IV administration of AAV8-TBG-p21 or AAV8-TBG-Null vectors. Same mice were used as in Figure 3. Control group received AAV8-TBG-Null and Luc mRNA-LNP injections, while the treated group received AAV8-TBG-p21 and HGF+EGF mRNA-LNP injections. **b** Quantification of percent p21^{+} hepatocytes, identified by morphology. At least 3 images were averaged per mouse, each image from a different liver lobe. Grey – control, green – treated group. **For all panels:** n=5 mice per group, each dot represents one mouse, error bars = SEM, scale = 100 μm, P values were calculated by unpaired two-sided student's t test, ns P > 0.05, * P ≤ 0.05, ** P ≤ 0.01, *** P ≤ 0.001, **** P ≤ 0.0001. Source data are provided as a Source Data file.

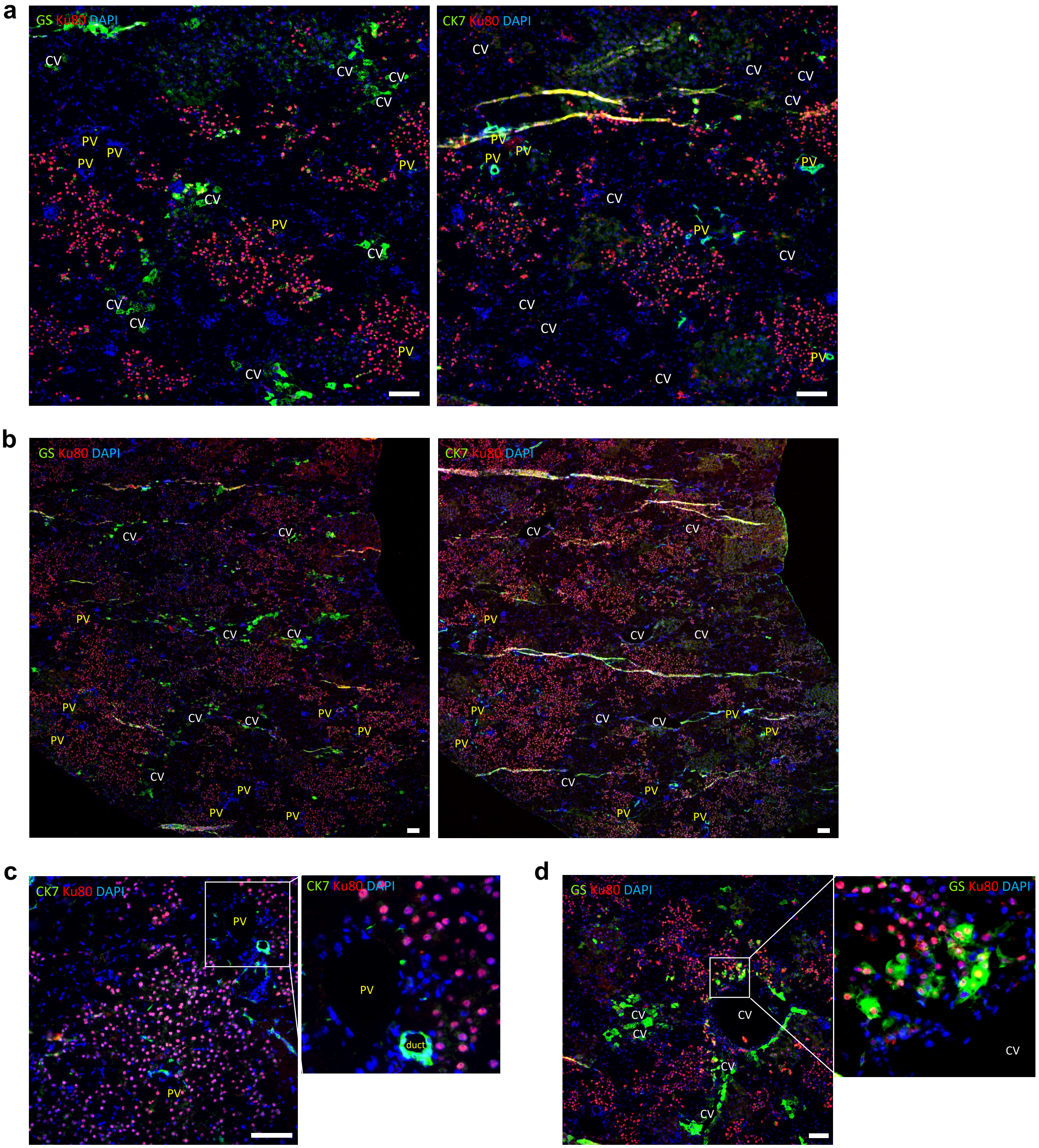

Supplementary Fig. 5 | Engrafted PHHs originate in the periportal region, span all liver zones, and adopt appropriate zonation markers. a Representative images of GS/Ku80 and CK7/Ku80 immunofluorescence stain on serial liver sections from experimental group 5 weeks post transplantation, highlighting an area of smaller clusters of engrafted PHHs. **b** Representative images of GS/Ku80 and CK7/Ku80 immunofluorescence stain on serial liver sections from experimental group 5 weeks post transplantation, highlighting an area of larger clusters of engrafted PHHs. **c** Representative image of CK7/Ku80 immunofluorescence stain on liver section from experimental group 5 weeks post transplantation, highlighting PHHs engrafted in periportal region. **d** Representative image of GS/Ku80 immunofluorescence stain on liver section from experimental group 5 weeks post transplantation, highlighting $GS^{+}Ku80^{+}$ PHHs engrafted in pericentral region. **For all panels:** Portal vein

(PV) regions are annotated in yellow, and central vein (CV) regions are annotated in white. n=5 mice, scale = 100 μm.

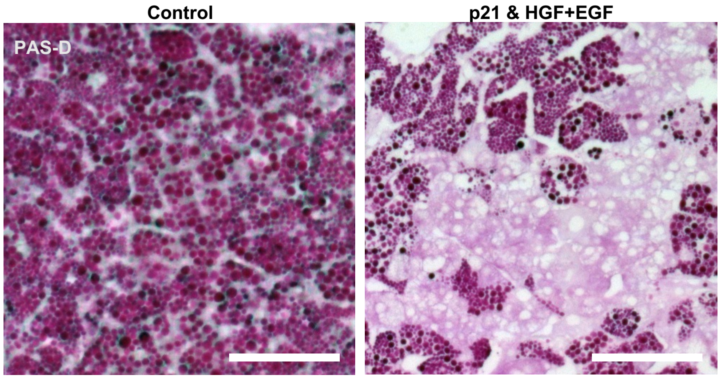

Supplementary Fig. 6 | PHH engraftment levels in experimental group are sufficient to reduce Z-AAT burden in the liver. Representative images of periodic acid Schiff - diastase (PAS-D) histology stain on liver sections from control and experimental groups 5 weeks post transplantation. hZ-AAT polymers are not digested by diastase, stain PAS-D$^+$, and are dark magenta in color. n=5 mice, scale = 100 μm.

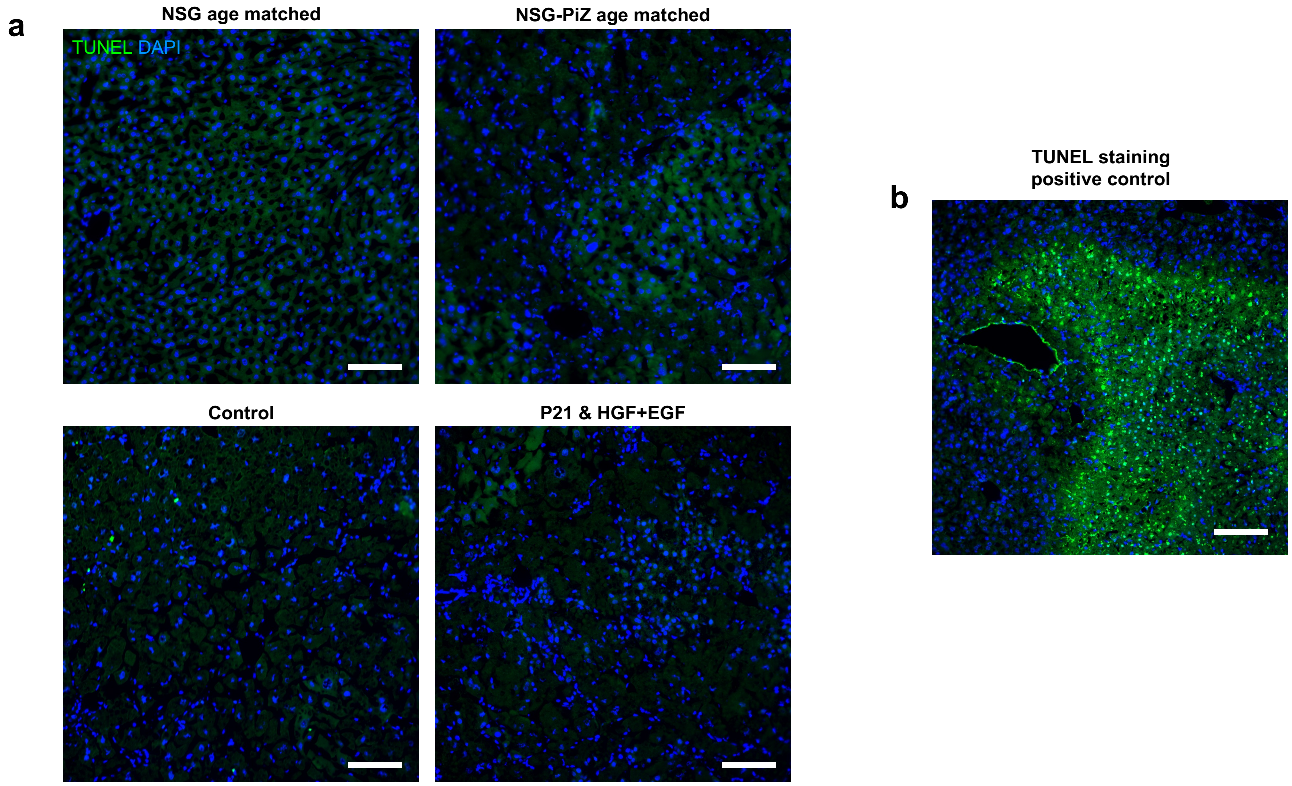

Supplementary Fig. 7 | Minimal hepatotoxicity is observed 5 weeks post transplantation after 10 mRNA-LNP injections. a. Representative images of fluorescence staining with TUNEL kit on liver tissue of control and treated groups 5 weeks post transplantation. Same mice were used as in Figure 3. NSG and NSG-PiZ age matched untreated controls are also shown. **b** Representative image of TUNEL staining on liver tissue section from APAP treated mouse, as a visual example of TUNEL^{+} staining. **For all panels:** For NSG n=3 mice, for NSG-PiZ n=3 mice, for both transplanted groups n=5 mice, scale = 100 μm.

Supplementary Table 1: DNA sequences to generate nucleoside-modified mRNA

Name	Sequence
Luciferase	ATGGAGGACGCCAAGAACATCAAGAAGGGCCCCGCCCCCTTCTACCCCCTGGA<br>GGACGGCACCGCCGGCGAGCAGCTGCACAAGGCCATGAAGCGgTACGCCCTG<br>GTGCCCGGCACCATCGCCTTCACCGACGCCCACATCGAGGTGGACATCACCTA<br>CGCCGAGTACTTCGAGATGTCCGTGCGCCTGGCCGAGGCCATGAAGCGgTACG<br>GCCTGAACACCAACCACCGCATCGTGGTGTGCTCCGAGAACTCCCTGCAGTTC<br>TTCATGCCCGTGCTGGGCGCCCTGTTCATCGGCGTGGCCGTGGCCCCCGCCA<br>ACGACATCTACAACGAGCGCGAGCTGCTGAACTCCATGGGCATCTCCCAGCCC<br>ACCGTGGTGTTCGTGTCCAAGAAGGGCCTGCAGAAGATCCTGAACGTGCAGAA<br>GAAGCTGCCCATCATCCAGAAGATCATCATCATGGACTCCAAGACCGACTACCA<br>GGGCTTCCAGTCCATGTACACCTTCGTGACCTCCCACCTGCCCCCCGGCTTCA<br>ACGAGTACGACTTCGTGCCCGAGTCCTTCGACCGCGACAAGACCATCGCCCTG<br>ATCATGAACTCCTCCGGCTCCACCGGCCTGCCCAAGGGCGTGGCCCTGCCCCA<br>CCGCACCGCCTGCGTGCGCTTCTCCCACGCCCGCGACCCCATCTTCGGCAACC<br>AGATCATCCCCGACACCGCCATCCTGTCCGTGGTGCCCTTCCACCACGGCTTC<br>GGCATGTTCACCACCCTGGGCTACCTGATCTGCGGCTTCCGCGTGGTGCTGAT<br>GTACCGCTTCGAGGAGGAGCTGTTCCTGCGCTCCCTGCAGGACTACAAGATCC<br>AGTCCGCCCTGCTGGTGCCCACCCTGTTCTCCTTCTTCGCCAAGTCCACCCTG<br>ATCGACAAGTACGACCTGTCCAACCTGCACGAGATCGCCTCCGGCGGCGCCCC<br>CCTGTCCAAGGAGGTGGGCGAGGCCGTGGCCAAGCGgTTCCACCTGCCCGGC<br>ATCCGCCAGGGCTACGGCCTGACCGAGACCACCTCCGCCATCCTGATCACCCC<br>CGAGGGCGACGACAAGCCCGGCGCCGTGGGCAAGGTGGTGCCCTTCTTCGAG<br>GCCAAGGTGGTGGACCTGGACACCGGCAAGACCCTGGGCGTGAACCAGCGCG<br>GCGAGCTGTGCGTGCGCGGCCCCATGATCATGTCCGGCTACGTGAACAACCCC<br>GAGGCCACCAACGCCCTGATCGACAAGGACGGCTGGCTGCACTCCGGCGACA<br>TCGCCTACTGGGACGAGGACGAGCACTTCTTCATCGTGGACCGCCTGAAGTCC<br>CTGATCAAGTACAAGGGCTACCAGGTGGCCCCCGCCGAGCTGGAGTCCATCCT<br>GCTGCAGCACCCCAACATCTTCGACGCCGGCGTGGCCGGCCTGCCCGACGAC<br>GACGCCGGCGAGCTGCCCGCCGCCGTGGTGGTGCTGGAGCACGGCAAGACC<br>ATGACCGAGAAGGAGATCGTGGACTACGTGGCCTCCCAGGTGACCACCGCCAA<br>GAAGCTGCGCGGCGGCGTGGTGTTCGTGGACGAGGTGCCCAAGGGCCTGACC<br>GGCAAGCTGGACGCCCGCAAGATCCGCGAGATCCTGATCAAGGCCAAGAAGG<br>GCGGCAAGATCGCCGTG
eGFP	ATGGTGAGCAAGGGCGAGGAGCTGTTCACCGGGGTGGTGCCCATCCTGGTCG<br>AGCTGGACGGCGACGTAAACGGCCACAAGTTCAGCGTGTCCGGCGAGGGCGA<br>GGGCGATGCCACCTACGGCAAGCTGACCCTGAAGTTCATCTGCACCACCGGCA<br>AGCTGCCCGTGCCCTGGCCCACCCTCGTGACCACCCTGACCTACGGCGTGCA<br>GTGCTTCAGCCGCTACCCCGACCACATGAAGCAGCACGACTTCTTCAAGTCCG<br>CCATGCCCGAAGGCTACGTCCAGGAGCGCACCATCTTCTTCAAGGACGACGGC<br>AACTACAAGACCCGCGCCGAGGTGAAGTTCGAGGGCGACACCCTGGTGAACC<br>GCATCGAGCTGAAGGGCATCGACTTCAAGGAGGACGGCAACATCCTGGGGCAC<br>AAGCTGGAGTACAACTACAACAGCCACAACGTCTATATCATGGCCGACAAGCAG<br>AAGAACGGCATCAAGGTGAACTTCAAGATCCGCCACAACATCGAGGACGGCAG<br>CGTGCAGCTCGCCGACCACTACCAGCAGAACACCCCCATCGGCGACGGCCCC<br>GTGCTGCTGCCCGACAACCACTACCTGAGCACCCAGTCCGCCCTGAGCAAAGA<br>CCCCAACGAGAAGCGCGATCACATGGTCCTGCTGGAGTTCGTGACCGCCGCCG<br>GGATCACTCTCGGCATGGACG AGCTGTACAAG

HGF	ATGTGGGTGACCAAGCTGCTGCCCGCCCTGCTGCTGCAGCACGTGCTGCTGCA<br>CCTGCTGCTGCTGCCCATCGCCATCCCCTACGCCGAGGGCCAGCGCAAGCGC<br>CGCAACACCATCCACGAGTTCAAGAAGTCCGCCAAGACCACCCTGATCAAGATC<br>GACCCCGCCCTGAAGATCAAGACCAAGAAGGTGAACACCGCCGACCAGTGCG<br>CCAACCGCTGCACCCGCAACAAGGGCCTGCCCTTCACCTGCAAGGCCTTCGTG<br>TTCGACAAGGCCCGCAAGCAGTGCCTGTGGTTCCCCTTCAACTCCATGTCCTCC<br>GGCGTGAAGAAGGAGTTCGGCCACGAGTTCGACCTGTACGAGAACAAGGACTA<br>CATCCGCAACTGCATCATCGGCAAGGGCCGCTCCTACAAGGGCACCGTGTCCA<br>TCACCAAGTCCGGCATCAAGTGCCAGCCCTGGTCCTCCATGATCCCCCACGAG<br>CACTCCTTCCTGCCCTCCTCCTACCGCGGCAAGGACCTGCAGGAGAACTACTG<br>CCGCAACCCCCGCGGCGAGGAGGGCGGCCCCTGGTGCTTCACCTCCAACCCC<br>GAGGTGCGCTACGAGGTGTGCGACATCCCCCAGTGCTCCGAGGTGGAGTGCAT<br>GACCTGCAACGGCGAGTCCTACCGCGGCCTGATGGACCACACCGAGTCCGGC<br>AAGATCTGCCAGCGgTGGGACCACCAGACCCCCCACCGCCACAAGTTCCTGCC<br>CGAGCGgTACCCCGACAAGGGCTTCGACGACAACTACTGCCGCAACCCCGACG<br>GCCAGCCCCGCCCCTGGTGCTACACCCTGGACCCCCACACCCGCTGGGAGTA<br>CTGCGCCATCAAGACCTGCGCCGACAACACCATGAACGACACCGACGTGCCCC<br>TGGAGACCACCGAGTGCATCCAGGGCCAGGGCGAGGGCTACCGCGGCACCGT<br>GAACACCATCTGGAACGGCATCCCCTGCCAGCGgTGGGACTCCCAGTACCCCC<br>ACGAGCACGACATGACCCCCGAGAACTTCAAGTGCAAGGACCTGCGCGAGAAC<br>TACTGCCGCAACCCCGACGGCTCCGAGTCCCCCTGGTGCTTCACCACCGACCC<br>CAACATCCGCGTGGGCTACTGCTCCCAGATCCCCAACTGCGACATGTCCCACG<br>GCCAGGACTGCTACCGCGGCAACGGCAAGAACTACATGGGCAACCTGTCCCAG<br>ACCCGCTCCGGCCTGACCTGCTCCATGTGGGACAAGAACATGGAGGACCTGCA<br>CCGCCACATCTTCTGGGAGCCCGACGCCTCCAAGCTGAACGAGAACTACTGCC<br>GCAACCCCGACGACGACGCCCACGGCCCCTGGTGCTACACCGGCAACCCCCT<br>GATCCCCTGGGACTACTGCCCCATCTCCCGCTGCGAGGGCGACACCACCCCCA<br>CCATCGTGAACCTGGACCACCCCGTGATCTCCTGCGCCAAGACCAAGCAGCTG<br>CGCGTGGTGAACGGCATCCCCACCCGCACCAACATCGGCTGGATGGTGTCCCT<br>GCGCTACCGCAACAAGCACATCTGCGGCGGCTCCCTGATCAAGGAGTCCTGGG<br>TGCTGACCGCCCGCCAGTGCTTCCCCTCCCGCGACCTGAAGGACTACGAGGC<br>CTGGCTGGGCATCCACGACGTGCACGGCCGCGGCGACGAGAAGTGCAAGCAG<br>GTGCTGAACGTGTCCCAGCTGGTGTACGGCCCCGAGGGCTCCGACCTGGTGC<br>TGATGAAGCTGGCCCGCCCCGCCGTGCTGGACGACTTCGTGTCCACCATCGAC<br>CTGCCCAACTACGGCTGCACCATCCCCGAGAAGACCTCCTGCTCCGTGTACGG<br>CTGGGGCTACACCGGCCTGATCAACTACGACGGCCTGCTGCGCGTGGCCCACC<br>TGTACATCATGGGCAACGAGAAGTGCTCCCAGCACCACCGCGGCAAGGTGACC<br>CTGAACGAGTCCGAGATCTGCGCCGGCGCCGAGAAGATCGGCTCCGGCCCCT<br>GCGAGGGCGACTACGGCGGCCCCCTGGTGTGCGAGCAGCACAAGATGCGCAT<br>GGTGCTGGGCGTGATCGTGCCCGGCCGCGGCTGCGCCATCCCCAACCGCCCC<br>GGCATCTTCGTGCGCGTGGCCTACTACGCCAAGTGGATCCACAAGATCATCCTG<br>ACCTACAAGGTGCCCCAGTCCTAA
EGF (secreted)	ATGGCCACCGGCTCCCGCACCTCCCTGCTGCTGGCCTTCGGCCTGCTGTGCCT<br>GCCCTGGCTGCAGGAGGGCTCCGCCATGAACTCCGACTCCGAGTGCCCCCTG<br>TCCCACGACGGCTACTGCCTGCACGACGGCGTGTGCATGTACATCGAGGCCCT<br>GGACAAGTACGCCTGCAACTGCGTGGTGGGCTACATCGGCGAGCGgTGCCAGT<br>ACCGCGACCTGAAGTGGTGGGAGCTGCGCTAA

Supplementary Table 2: List of antibodies

Antibody	Company	Catalog #	Host Species	Application	Dilution	Notes
GFP	Invitrogen	A10262	Chicken	IF-Fr	1:300	-
HGF	R&D	AF-294	Goat	IF-Fr	1:20	-
EGF	R&D	MAB236	Mouse	IF-Fr	1:30	M.O.M. Kit (Vector Labs BMK-2202)
P21	Abcam	ab188224	Rabbit	IF-Fr	1:500	0.2% triton X with primary antibody incubation
hALB	Bethyl	A80-129A	Goat	IF-Fr	1:1000	-
Ku80	Cell Signaling	mAb #2180	Rabbit	IF-Fr	1:50	0.2% triton X with primary antibody incubation
2C1	Hycult Biotech	HM2289	Mouse	IF-Fr	1:50	M.O.M. Kit (Vector Labs BMK-2202)
GS	Invitrogen	PA581398	Rabbit	IF-Fr	1:500	For use with Ku80, must do intermediate blocking step with FAB Donkey Anti-Rabbit IgG (H+L)
CK7	Abcam	ab181598	Rabbit	IF-Fr	1:500	For use with Ku80, must do intermediate blocking step with FAB Donkey Anti-Rabbit IgG (H+L)

IF-Fr: immunofluorescence, frozen sections
