# Supplementary material for: Transient growth factor expression via mRNA in lipid nanoparticles promotes hepatocyte cell therapy in mice
Source: Nat Commun. 2024 Jun 12;15:5010. doi: 10.1038/s41467-024-49332-8 (PMC11169405; doi:10.1038/s41467-024-49332-8)
Supplement: Supplementary file 3 — Reporting Summary [file 41467_2024_49332_MOESM3_ESM.pdf]

nature portfolio

Corresponding author(s): Valerie Gouon-Evans

Last updated by author(s): May 19, 2024

Reporting Summary

Nature Portfolio wishes to improve the reproducibility of the work that we publish. This form provides structure for consistency and transparency in reporting. For further information on Nature Portfolio policies, see our Editorial Policies and the Editorial Policy Checklist.

Statistics

For all statistical analyses, confirm that the following items are present in the figure legend, table legend, main text, or Methods section.

n/a	Confirmed	
☐	☒	The exact sample size (n) for each experimental group/condition, given as a discrete number and unit of measurement
☐	☒	A statement on whether measurements were taken from distinct samples or whether the same sample was measured repeatedly
☐	☒	The statistical test(s) used AND whether they are one- or two-sided<br>*Only common tests should be described solely by name; describe more complex techniques in the Methods section.*
☐	☒	A description of all covariates tested
☒	☐	A description of any assumptions or corrections, such as tests of normality and adjustment for multiple comparisons
☐	☒	A full description of the statistical parameters including central tendency (e.g. means) or other basic estimates (e.g. regression coefficient) AND variation (e.g. standard deviation) or associated estimates of uncertainty (e.g. confidence intervals)
☒	☐	For null hypothesis testing, the test statistic (e.g. F, t, r) with confidence intervals, effect sizes, degrees of freedom and P value noted<br>*Give P values as exact values whenever suitable.*
☒	☐	For Bayesian analysis, information on the choice of priors and Markov chain Monte Carlo settings
☒	☐	For hierarchical and complex designs, identification of the appropriate level for tests and full reporting of outcomes
☒	☐	Estimates of effect sizes (e.g. Cohen's d, Pearson's r), indicating how they were calculated

Our web collection on statistics for biologists contains articles on many of the points above.

Software and code

Policy information about availability of computer code

Data collection

Each group of mice included at least 5 mice. This information was written in each figure legend.

Data analysis

All statistical analyses were performed using GraphPad Prism version 10.0.0 for Mac, GraphPad Software, Boston, Massachusetts USA, www.graphpad.com. For comparison between two groups, an unpaired t-test was used to calculate statistical significance. For comparing multiple groups, an ordinary one-way unpaired ANOVA was performed. Quantitative data are shown as mean ± standard error of the mean (SEM) and are considered statistically significant when p<0.05 (ns P > 0.05, * P ≤ 0.05, ** P ≤ 0.01, *** P ≤ 0.001, **** P ≤ 0.0001).

All images were taken using a Nikon microscope (Nikon Eclipse Ni-E) and analyzed using NIS Elements software and Fiji. For all histology quantification, measurements for each mouse were averaged using three 10X images per mouse each from a different liver lobe. For quantifying EdU+ or p21+hepatocytes, positive cells were counted manually based on morphology using high power magnification. To quantify the number of PHH clusters/field, cells/cluster, and EdU+ cells/cluster, positive events were manually counted per image using high power magnification. To quantify the percent hALB+ or 2C1+ area, positive areas were measured using Fiji and were divided by the total tissue area, accounting for empty areas due to large vasculature such as portal and central veins. The percentage of liver repopulation was quantified by tracing the area occupied by hALB+Ku80+ PHHs and dividing by total tissue area, accounting for empty areas due to large vasculature such as portal and central veins.

For manuscripts utilizing custom algorithms or software that are central to the research but not yet described in published literature, software must be made available to editors and reviewers. We strongly encourage code deposition in a community repository (e.g. GitHub). See the Nature Portfolio guidelines for submitting code & software for further information.

Data

Policy information about availability of data

All manuscripts must include a data availability statement. This statement should provide the following information, where applicable:

- Accession codes, unique identifiers, or web links for publicly available datasets
- A description of any restrictions on data availability
- For clinical datasets or third party data, please ensure that the statement adheres to our policy

All data generated and analyzed during this study are included in this published article and its Supplementary Information. Source data are provided as a Source Data file. Sequences are included in Supplementary Information.

Research involving human participants, their data, or biological material

Policy information about studies with human participants or human data. See also policy information about sex, gender (identity/presentation), and sexual orientation and race, ethnicity and racism.

Reporting on sex and gender	This study does not involve human participants.
Reporting on race, ethnicity, or other socially relevant groupings	*Please specify the socially constructed or socially relevant categorization variable(s) used in your manuscript and explain why they were used. Please note that such variables should not be used as proxies for other socially constructed/relevant variables (for example, race or ethnicity should not be used as a proxy for socioeconomic status).* *Provide clear definitions of the relevant terms used, how they were provided (by the participants/respondents, the researchers, or third parties), and the method(s) used to classify people into the different categories (e.g. self-report, census or administrative data, social media data, etc.)* *Please provide details about how you controlled for confounding variables in your analyses.*
Population characteristics	*Describe the covariate-relevant population characteristics of the human research participants (e.g. age, genotypic information, past and current diagnosis and treatment categories). If you filled out the behavioural & social sciences study design questions and have nothing to add here, write "See above."*
Recruitment	*Describe how participants were recruited. Outline any potential self-selection bias or other biases that may be present and how these are likely to impact results.*
Ethics oversight	*Identify the organization(s) that approved the study protocol.*

Note that full information on the approval of the study protocol must also be provided in the manuscript.

Field-specific reporting

Please select the one below that is the best fit for your research. If you are not sure, read the appropriate sections before making your selection.

[x] Life sciences [ ] Behavioural & social sciences [ ] Ecological, evolutionary & environmental sciences

For a reference copy of the document with all sections, see nature.com/documents/nr-reporting-summary-flat.pdf

Life sciences study design

All studies must disclose on these points even when the disclosure is negative.

Sample size	We used at least 5 mice per group in each experiment based on previous publications using the same injury models and assays to characterized the liver injury. The sample size was shown to be sufficient to identify statistical differences between groups for the specific assays used. All group sizes are indicated in each figure legends.
Data exclusions	No data were excluded
Replication	All in vivo experiments were performed with at least 5 mice per group and per time point. For all histology quantification, measurements for each mouse were averaged using three 10X images per mouse each from a different liver lobe. "N" were defined as numbers of mice.
Randomization	Mice used in this study were inbred, all the same age, the same sex, and therefore about the same weight. Mice were provided by the Jackson laboratory. All animal studies were approved by the Boston University IACUC and were consistent with local, state, and federal regulations as applicable.
Blinding	Tissue staining were analyzed by 2 to 3 blinded authors (ARS, AA, and SW) and the identity of the sample group was unknown when tissue were analyzed. Liver tissue staining or immunostaining-based data were quantified and supported by analyses of serum data from the same mice.

Reporting for specific materials, systems and methods

We require information from authors about some types of materials, experimental systems and methods used in many studies. Here, indicate whether each material, system or method listed is relevant to your study. If you are not sure if a list item applies to your research, read the appropriate section before selecting a response.

Materials & experimental systems

n/a	Involved in the study	
☐	☒	Antibodies
☒	☐	Eukaryotic cell lines
☒	☐	Palaeontology and archaeology
☐	☒	Animals and other organisms
☒	☐	Clinical data
☒	☐	Dual use research of concern
☒	☐	Plants

Methods

n/a	Involved in the study	
☒	☐	ChIP-seq
☒	☐	Flow cytometry
☒	☐	MRI-based neuroimaging

Antibodies

Antibodies used	All antibodies used in this study have been listed in the table in the supplementary data file.
Validation	We validated specificity of each antibody for immuno-staining by using the corresponding isotype negative controls.

Animals and other research organisms

Policy information about studies involving animals; ARRIVE guidelines recommended for reporting animal research, and Sex and Gender in Research

Laboratory animals	In this study, we used 2 mouse colonies purchased from the Jackson Laboratory. For chronic injury, the transgenic NOD.Cg-Prkdcscid Il2rgtm1Wjl Tg(SERPINA1*E342K)#Slcw/SzJ (NSG-PiZ) colony was used, mimicking human alpha-1 antitrypsin deficiency (AATD) associated liver disease. NSG-PiZ mice are homozygous for a transgene expressing the human mutated PiZ allele. For acute liver injury, immunodeficient NOD.Cg-Prkdcscid Il2rgtm1Wjl/SzJ (NSG) mice were used.
Wild animals	N/A
Reporting on sex	All mice used in this study were male as males have been reported to develop a more severe liver disease in both the PiZ chronic model and the acetaminophen overdose-acute model.
Field-collected samples	N/A
Ethics oversight	All animal studies were approved by the Boston University IACUC and were consistent with local, state, and federal regulations as applicable

Note that full information on the approval of the study protocol must also be provided in the manuscript.

Plants

Seed stocks	*Report on the source of all seed stocks or other plant material used. If applicable, state the seed stock centre and catalogue number. If plant specimens were collected from the field, describe the collection location, date and sampling procedures.*
Novel plant genotypes	*Describe the methods by which all novel plant genotypes were produced. This includes those generated by transgenic approaches, gene editing, chemical/radiation-based mutagenesis and hybridization. For transgenic lines, describe the transformation method, the number of independent lines analyzed and the generation upon which experiments were performed. For gene-edited lines, describe the editor used, the endogenous sequence targeted for editing, the targeting guide RNA sequence (if applicable) and how the editor was applied.*
Authentication	*Describe any authentication procedures for each seed stock used or novel genotype generated. Describe any experiments used to assess the effect of a mutation and, where applicable, how potential secondary effects (e.g. second site T-DNA insertions, mosiacism, off-target gene editing) were examined.*
